# In paired preference tests, domestic chicks innately choose the colour green over red, and the shape of a frog over a sphere when both stimuli are green

**DOI:** 10.1007/s10071-023-01821-x

**Published:** 2023-08-23

**Authors:** Francesca Protti-Sánchez, Uwe Mayer, Hannah M. Rowland

**Affiliations:** 1 Max Planck Research Group Predators and Toxic Prey, Max Planck Institute for Chemical Ecology, Hans Knöll Straße 8, 07745 Jena, Germany; 2 Center for Mind/Brain Sciences (CIMeC), University of Trento, Piazza Manifattura 1, 38068 Rovereto, TN Italy

**Keywords:** Aposematism, Foraging bias, Quinine, Receiver psychology, *Gallus gallus*

## Abstract

Many animals express unlearned colour preferences that depend on the context in which signals are encountered. These colour biases may have evolved in response to the signalling system to which they relate. For example, many aposematic animals advertise their unprofitability with red warning signals. Predators’ innate biases against these warning colours have been suggested as one of the potential explanations for the initial evolution of aposematism. It is unclear, however, whether unlearned colour preferences reported in a number of species is truly an innate behaviour or whether it is based on prior experience. We tested the spontaneous colour and shape preferences of dark-hatched, unfed, and visually naive domestic chicks (*Gallus gallus*). In four experiments, we presented chicks with a choice between either red (a colour typically associated with warning patterns) or green (a colour associated with palatable cryptic prey), volume-matched spheres (representing a generalised fruit shape) or frogs (representing an aposematic animal’s shape). Chicks innately preferred green stimuli and avoided red. Chicks also preferred the shape of a frog over a sphere when both stimuli were green. However, no preference for frogs over spheres was present when stimuli were red. Male chicks that experienced a bitter taste of quinine immediately before the preference test showed a higher preference for green frog-shaped stimuli. Our results suggest that newly hatched chicks innately integrate colour and shape cues during decision making, and that this can be augmented by other sensory experiences. Innate and experience-based behaviour could confer a fitness advantage to novel aposematic prey, and favour the initial evolution of conspicuous colouration.

**Supplementary Information:**

The online version contains supplementary material available at 10.1007/s10071-023-01821-x.

## Introduction

Animals rely on colourful visual signals in a variety of contexts including mate attraction, territorial defence, social interactions, and predator avoidance (Cuthill et al. 2017). Some colours are very common across different taxa and geographical regions, despite their different functions. For example, red is used to attract mates by primates, fish, and crabs (Östlund-Nilsson et al. 2006; Baldwin and Johnsen 2009; Rigaill et al. 2019). Sticklebacks use red signals to defend territories (Kim and Velando 2014). Red is also a powerful filial imprinting stimulus for domestic chicks (Salzen et al. 1971; Miura et al. 2020). In a foraging context, red can be a positive or negative signal. In fruits, red signals profitability (Schaefer and Schaefer 2006; Albrecht et al. 2012), but in insects, it can signal unprofitability, as with aposematic prey (Majerus 2016). Herein lies a problem for naive signal receivers—how to distinguish between profitability and unprofitability when the colour signal is the same. Many animals express unlearned colour preferences that depend on the context in which signals are encountered (Salzen et al. 1971; Gamberale-Stille and Tullberg 2001; Zachar et al. 2008; Paluh et al. 2015; Miura et al. 2020). These colour biases may have evolved in response to the signalling system to which they relate (Guilford and Dawkins 1991; Endler 1992; Endler and Basolo 1998; Kokko et al. 2003).

A variety of aposematic prey have evolved red warning signals (e.g. ladybirds, heliconius butterflies, poison frogs, salamanders; Bezzerides et al. 2007; Finkbeiner et al. 2014; Kraemer et al. 2015; Saporito et al. 2007), which predators learn to associate with their unprofitability (Ruxton et al. 2019). Given that these conspicuous colours can attract the attention of naive potential predators that have yet to learn about the link between unprofitability and conspicuousness, the initial evolution of aposematism has been described as paradoxical (Mappes et al. 2005). Neophobia, dietary wariness, and social learning have been proposed to explain how prey overcome the initial high costs of conspicuousness (Mappes et al. 2005; Marples et al. 2005; Marples and Mappes 2010; Lee et al. 2010; Hämäläinen et al. 2021). Innate biases against such colours have also been predicted to aid the origin of aposematism (Lindström 1999). Some predators avoid colours and patterns typical of aposematic chemically defended prey, despite having never encountered the prey before (Caldwell & Rubinoff 1983; Smith 1975, 1977).

For instance, wild-caught, young blackcaps (*Sylvia atricapilla*) and domestic chicks prefer green over red insects (Gamberale-Stille and Tullberg 2001; Gamberale-Stille et al. 2007). Hand-reared juvenile blackcaps also express an unlearned preference for red fruits, which is absent in adults (Schmidt and Schaefer 2004). However, there is some inconsistency in measured unlearned colour preferences. For example, young blackcaps and domestic chicks show no colour preference between red or green fruits (Gamberale-Stille et al. 2007; Zachar et al. 2008). Great tinamous prefer red spherical stimuli over red frog-shaped models but show no preference for differently coloured frog stimuli (Paluh et al. 2015). This inconsistency could be because colour responses depend on an interaction between colour and shape (e.g. prey vs fruits), and the context in which they are encountered (Kuenzinger et al. 2019).

Inconsistences in unlearned colour preferences could also be explained by prior experience of the animals tested, who, in most cases, had experienced food, social partners, parents, etc., before their colour and shape preferences were tested (e.g. Mastrota and Mench 1995; Roper 1990; Roper and Cook 1989; Zachar et al. 2008). This is important because colour preferences can depend on an animal’s early experience (Lindström et al. 1999; Schmidt and Schaefer 2004; Teichmann et al. 2020) and can be rapidly updated by other sensory experiences, i.e. newly hatched chicks can learn the association between colour and bitter taste in a single trial (Rose 2000; Tiunova et al. 2020).

Experiencing a novel sound, odour, or bitter taste can also cause predators to increase their bias against novel foods or foods with visual traits typically associated with aposematism, such as conspicuousness, or a red or yellow colour (Marples and Roper 1996; Rowe and Guilford 1996, 1999b, 1999a; Jetz et al. 2001; Lindström et al. 2001; Rowe and Skelhorn 2005; Skelhorn et al. 2008; Siddall and Marples 2008, 2011; Skelhorn 2011). Therefore, when investigating colour preferences, it is crucial to distinguish between which components of behaviour are innate and which are experience dependent.

To test this, we investigated the colour and shape preferences of newly hatched, visually naive and unfed domestic chicks (*Gallus gallus domesticus*). In four experiments, we presented chicks with a choice between either red (a colour typically associated with warning patterns) or green (a colour associated with palatable cryptic prey), volume-matched spheres (representing a generalised fruit shape) or frogs (representing an aposematic animal’s shape). We chose the frog and sphere shapes for our stimuli because these represent ecologically relevant shapes for chickens. Chickens are omnivorous, with a natural diet that includes fruits, grains, and other animals such as invertebrate and vertebrate prey (Klasing 2005). Chickens have also been used as model predators to study aposematism in insects (Gamberale-Stille and Tullberg 1999; Gamberale-Stille 2001; Hauglund et al. 2006) and poison frogs (Darst and Cummings 2006; Darst et al. 2006; Amézquita et al. 2013; Lawrence and Noonan 2018). But their innate response to frogs (unlike insects) has not previously been tested, leaving an open question of whether early foraging biases are limited to a specific prey category.

**Fig. 1:**
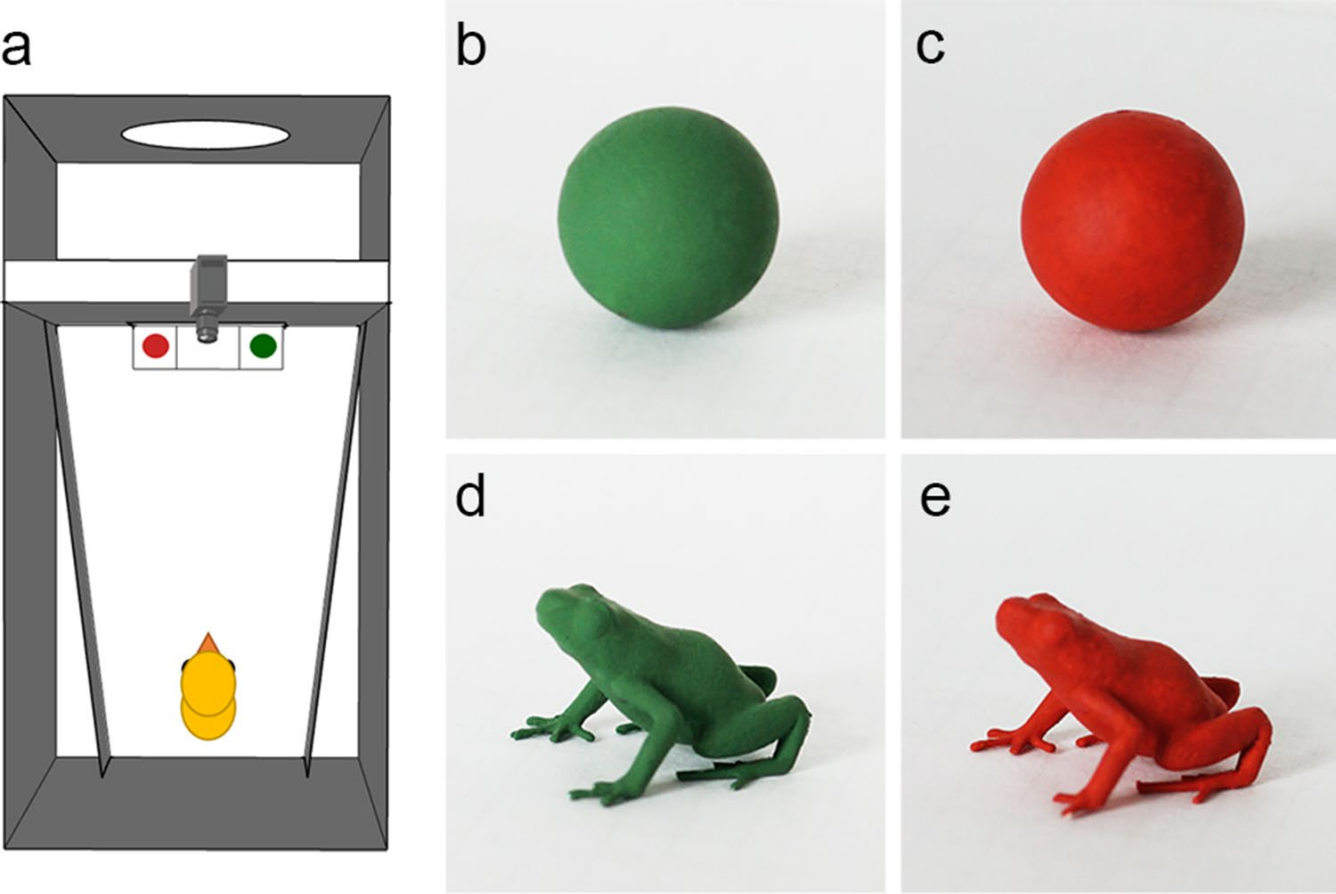
Experimental setup. **a** Schematic view from above of the test arena with the green and red spheres. **b**–**e** Photos of the four stimuli

Many of the behavioural responses that domestic chicks show towards aposematic animals have also been documented in a range of bird species (Smith 1975, 1977; Caldwell and Rubinoff 1983; Mastrota and Mench 1995; Lindström et al. 1999). Investigating domestic chicks’ innate behaviours can be useful for understanding the mechanisms of behaviour (Rosa Salva et al. 2015, 2018, 2019; Di Giorgio et al. 2017, 2023; Lemaire et al. 2022). Domestic chicks are an especially useful model organism because they are a nidifugous species that soon after hatching move around and spontaneously peck on small objects. The in-egg development of chicks also allows for strictly controlling pre- and post-hatching experiences. In our experiments, the eggs were incubated and hatched in dark incubators and then taken directly from the dark incubators to participate in a choice test between different pairs of stimuli (Fig. 1). This allows us to be certain that the expressed behaviours are truly innate.

In each experiment, we provided a chick with a paired choice test between red and green spheres, red and green frogs, green frogs and green spheres, or red frogs and red spheres. We predicted that frog shape would increase the avoidance of red but not green, because the combination of frog shape and red colour should increase the perceived risk of toxicity compared to a green frog. We also predicted that chicks would be less willing to attack red frogs than spheres, revealing innate avoidance of warning colour-shape combinations. To determine whether experiencing a bitter taste would affect or induce any foraging bias (Rowe and Skelhorn 2005; Skelhorn and Rowe 2005; Skelhorn et al. 2008), half of the animals were presented with a bitter taste (quinine) before the preference test in all experiments, whereas the other half was given water. We predicted that experiencing a bitter taste before encountering the stimuli would bias chicks’ attacks from red to green, as reported in previous studies (Rowe and Skelhorn 2005; Skelhorn and Rowe 2005; Skelhorn et al. 2008). The combination of choice tests and taste treatments were designed to tease apart which components of chicks’ responses to colour and shape are innate and which are dependent on other sensory experience.

## Methods

### Subjects

We used 175 newly hatched domestic chicks (*Gallus gallus domesticus*) of the Aviagen ROSS 308 strain. Fertilised eggs were obtained from a commercial hatchery (CRESCENTI Società Agricola S.r.l. –Allevamento Trepola–cod. Allevamento127BS105/2). Eggs were incubated and hatched within dark incubators (Marans P140TU-P210TU) at 37.7 °C, with 60% humidity in a dark room.

### Experimental setup

The experimental cage consisted of a metal rectangle (28 × 40 × 32 cm; *W* × *L* × *H*) with a circular opening at one end (Fig. 1a). We covered the inside walls and the floor with white polypropylene sheets (Poliplak). We divided the cage into two compartments with a white polypropylene wall 17 cm (hereafter referred to as an occluder) from the main opening of the cage. This was done to create a small area where the stimuli could be kept out of sight of the chicks before presenting the stimuli to the chicks in the larger experimental area. The occluder had an opening of 14 × 5 cm (*L* × *W*) in the middle that was covered with a movable piece of the same material to allow the insertion of the stimuli during the experiment. This also provided a white background against which the stimuli would be presented to the chicks. Two smaller internal walls were added within the experimental arena creating a trapezoid area to encourage chicks to approach the stimuli rather than explore the corners in the back of the cage. Chicks’ behaviours were recorded directly to a computer using a webcam (Microsoft LifeCam Cinema for Business) positioned above the separating wall. The arena was illuminated by a Philips classic tone 60 W light bulb placed 57 cm above the cage floor. The lamp provided a homogeneous illumination of the experimental cage without shades. The irradiance spectrum of the lamp is shown in Fig. S1.

### Stimuli

We obtained 3D-printed spherical and frog-shaped stimuli from SaviMade (Windsor, Ontario, Canada). Spheres had a diameter of 11.45 mm and a volume of 785.99 mm^3^ (Fig. 1b, c). The frog stimuli were based on the morphology of the strawberry poison frog (*Oophaga pumilio,* Dendrobatidae) a species known to be aposematic (Saporito et al. 2007; Paluh et al. 2013; Rojas et al. 2018), with a snout-to-vent length (SVL) of 19.5 mm (based on the mean SVL of a sampled population at La Selva, Sarapiqui, Costa Rica; Protti-Sánchez et al. unpublished) and a volume of 786.463 mm^3^ (Fig. 1d, e). We thus matched the volume of the stimuli (~ 99.9%) to reduce the likelihood that volume differences in the stimuli could explain the chicks’ responses. Both frog-shaped and sphere-like stimuli have been readily attacked by wild birds in field experiments (Saporito et al. 2007; Paluh et al. 2013, 2015).

We painted the stimuli either red or green using Vallejo red (“dark vermillion 70.947”) and green (“intermediate green 70.891”) acrylic paint. We ensured that both green and red were equally bright according to the chick’s visual system, so only the hue was manipulated (see Fig. S2 and Table S1 for visual modelling results). We measured the stimuli with a QR200-7-UV-BX bifurcal optical fibre connected to an Ocean Optics FLAME-S-XR1-ES spectrometer and a PX-2 Pulsed Xenon light source. We used a WS1 white reflectance standard for calibration. We recorded reflectance spectra using the software Ocean View v. 1.6.7 2013 from Ocean Optics (Fig. S3). We also measured the reflectance spectra of the white polypropylene sheets (Poliplak) background of the experimental cage where the stimuli were presented to the chicks. We calculated the colour (∆S) and brightness (∆L) contrast between the two paint colours and between each paint colour and the background using the Vorobyev–Osorio colour discrimination model, which is based on evidence that colour discrimination is determined by noise arising in the photoreceptors and is independent of light intensity (Vorobyev and Osorio 1998). We calculated the visual contrasts using the package pavo2 (Maia et al. 2019) in R Studio v. 4.2.2 (R Core Team 2022) and the visual system of domestic chicken for chromatic (longwave, LWS, *λ*max 418 nm; mediumwave, MWS, *λ*max 455 nm; shortwave, SWS, λmax 508 nm; ultraviolet, UVS, λmax 570 nm; Hart 2001) and achromatic contrast (achromatic = ch.dc, based on chicken double-cone). We used a Weber fraction value of 0.06 (Olsson et al. 2015) for the most abundant cone type and 0.36 for the achromatic contrast based on the average of brightness discrimination from Olsson et al. (2015). The density of photoreceptors was *n* = 1, 1.5, 2.5, 2 (Olsson et al. 2016). We used the irradiance spectra measured inside the cages with a UPRtek PG100N PAR spectrometer (Fig. S1).

We attached the painted stimuli with hot glue to white polypropylene rectangles (4 × 3.5 cm), which were then attached to a bigger rectangle (14 × 3.5 cm) as a base to maintain both stimuli with the same separation. The distance between both stimuli remained constant over trials at 10 cm.

### Experimental procedure

To test innate colour preference, we presented chicks with either two spheres resembling a fruit (green and red, *n* = 44) or two frogs (green and red, *n* = 42). To test innate shape preference, we presented chicks with either a green sphere and frog (*n* = 42) or a red sphere and frog (*n* = 47).

Chicks were caught in the dark on the day of hatching, placed in a fully covered small black box, and taken to the experimental room (average temperature = 23 °C). There is no evidence that chicks are impaired either on emergence or subsequently after having been kept in the dark (Ham and Osorio 2007). Chicks were randomly assigned to a taste treatment (bitter taste or control). The chick was removed from the box, and their eyes were covered by the researcher’s hand (FPS). The chicks were given 5 ml of the experimental taste with a 10 ml plastic pipette. Each chick received either tap water or 10 mM quinine dissolved in water (Alfa Aesar A10459 99%). This concentration is the same as that used in a previous study showing clear aversive behaviours in four-day-old female chicks of the same strain (Protti-Sánchez et al. 2022). Chicks also react with disgust to quinine on the first day after hatching (Ganchrow et al. 1990). Both tastes were kept at room temperature, and a different pipette was used for each experimental taste. Immediately after receiving the liquid, chicks were placed in the experimental arena's releasing site, facing the occluder and at the back of the arena (Fig. 1a). Once in the arena, chicks were given 1 min to habituate, after which the occluder was lifted. The stimuli were presented with a fast left–right movement for 5 s to stimulate the chick’s attention towards the stimuli. Video recording started at this point. Chicks were then given 6 min to approach and peck at the stimuli. Once a chick pecked at either stimulus, it was given a further 2 min. This was to ensure that all chicks had the same time with the stimuli after the first peck. Stimulus position (left–right) was changed in each trial to remove the effect of any side bias chicks might have (Vallortigara et al. 1988, 1999; Morandi-Raikova et al. 2020). After the experiment, chicks were removed from the experimental arena, sexed and returned to the animal facilities.

### Video analysis

Videos were analysed with the software Boris v.7.12.2 (Friard and Gamba 2016), and coders were blind to the taste chicks received. We recorded which stimulus was first pecked by the chicks and how many times they pecked at each stimulus for 2 min after the first peck. Both variables are a measure of preference.

### Statistical analysis

All statistical analyses were conducted and plots were made using R studio v. 4.2.2 (R Core Team 2022). To test whether the probability of choosing a stimulus first (i.e. green vs red or sphere vs frog) depends on the taste received and sex of the chick, we converted the first choice response to a binomial variable, with 1 indicating a first choice for green/sphere, and zero a first choice for red/frog. We then used a Generalised Linear Model (GLM) with binomial error distribution, taste and sex as fixed effects, and the interaction between taste and sex. To test for the significance within this model, we used a Chi-square test based on log-likelihood ratios, using the function Anova of the car package (Fox and Weisberg 2018). If, with this model, the factors (taste and sex) had no effects and no interactions, we performed a Chi-square test to determine whether the probability of choosing a stimulus differed from random. In the presence of a significant interaction, we performed further GLM analyses with a binomial distribution, separately testing the effect of sex in the two taste groups (quinine and water) and the effect of taste on the two sexes.

To test whether taste and sex affected the total number of pecks on the stimuli, we used a GLM with quasibinomial error distribution, which considered the proportion of pecks to each stimulus. In addition, this model assessed the statistical significance of predictor variables with Chi-square tests based on log-likelihood ratios using the function Anova of the car package (Fox and Weisberg 2018). To test whether the number of pecks differed between stimuli we conducted paired t-tests, or Wilcoxon signed-rank tests if normality assumptions were not met.

## Results

### Colour preference

#### Green sphere vs. red sphere

**Fig. 2:**
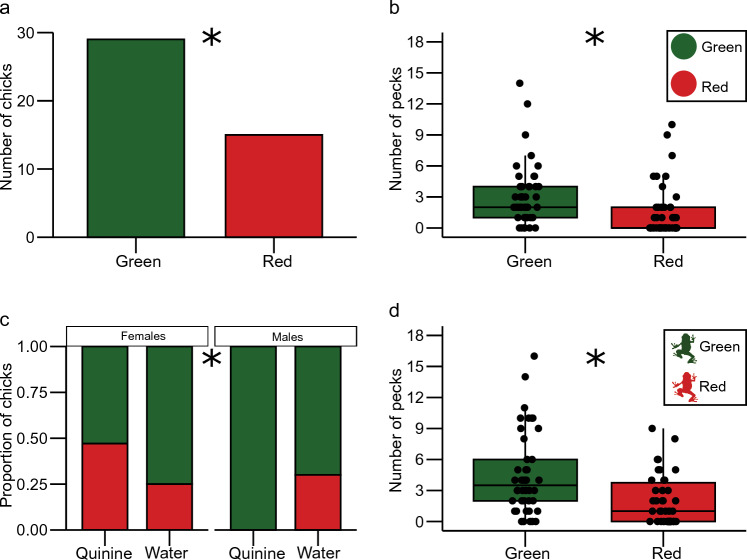
Colour preferences. **a** Number of chicks that directed their first choice to green and red spheres. **b** Total number of pecks at green and red spheres. **c** Proportion of chicks that directed their first choice to green and red frog, plotted separately for sex and taste treatment. The proportion of choices for green is plotted on the upper portion of each bar. **d** Total number of pecks at the green and red frog. *Statistically significant differences

There were no main effects of taste or sex on the chicks’ first pecks and no significant interaction between the two factors (Table 1). However, significantly more chicks pecked the green sphere before the red (29 of 44 chose green: *X*^2^ = 4.4545, *df* = 1, *p* = 0.035; Fig. 2a).

**Table 1:** Results of the GLM analyses for the first pecks (*p* < 0.05 is highlighted in bold)

Experiment	Factors	*X*^2^	*df*	*p*
Colour				
Green sphere vs. red sphere	Taste	0.008	1	0.928
	Sex	0.012	1	0.911
	Taste*sex	0.603	1	0.437
Green frog vs. red frog	Taste	0.001	1	0.979
	Sex	2.329	1	0.127
	Taste*sex	4.771	1	**0.029**
Shape				
Green sphere vs. green frog	Taste	0.151	1	0.697
	Sex	1.537	1	0.215
	Taste*sex	1.926	1	0.165
Red sphere vs. red frog	Taste	0.189	1	0.664
	Sex	0.420	1	0.517
	Taste*sex	0.106	1	0.744

There were no main effects of taste or sex on the number of pecks at red or green spheres (Table 2). Chicks directed a higher number of pecks at the green sphere than the red sphere (mean ± SEM, rounded numbers, green: 3.16 ± 0.4; red: 1.5 ± 0.4; V = 672.5, *p* < 0.002; Fig. 2b).

**Table 2:** Results of the GLM analyses for the total number of pecks

Experiment	Factors	*X*^2^	*df*	*p*
Colour				
Green sphere vs. red sphere	Taste	0.314	1	0.575
	Sex	0.050	1	0.822
	Taste*sex	0.535	1	0.464
Green frog vs. red frog	Taste	0.423	1	0.515
	Sex	0.370	1	0.543
	Taste*sex	0.416	1	0.519
Shape				
Green sphere vs. green frog	Taste	3.130	1	0.077
	Sex	0.338	1	0.561
	Taste*sex	0.069	1	0.793
Red sphere vs. red frog	Taste	2.712	1	0.1
	Sex	0.001	1	0.978
	Taste*sex	0.144	1	0.705

#### Green frog vs. red frog

A significant interaction between taste and sex affected the probability that a chick first pecked a green or red frog (Table 1). We first split the dataset by taste and performed a GLM on the animals that received quinine to test for the effect of sex in this group, which was significant (*X*^2^ = 7.04, *df* = 1, *p* = 0.007). A similar number of females pecked first at either the green or the red frog (9 out of 17 females chose green; *X*^2^ = 0.0588, *df* = 1, *p* = 0.808, Fig. 2c), whereas all seven males directed the first peck to the green frog, showing a significant preference for the green after receiving quinine (*X*^2^ = 7, *df* = 1, *p* = 0.008; Fig. 2c). Male and female chicks that received water did not differ in their first choice (*X*^2^ = 0,056, *df* = 1, *p* = 0.813, Fig. 2c). Second, we split the dataset by sex. Males that received quinine showed a preference for green frogs over red frogs compared to those that received water, but this was not significant at the alpha 0.05 level (*X*^2^ = 3.6267, *df* = 1, *p* = 0.057). Females showed no significant difference in preference if they received quinine or water (*X*^2^ = 1.1451, *df* = 1, *p* = 0.285). Overall, more chicks pecked first at the green than the red frog, but this was not significant at the alpha 0.05 level (13 out of 18 chose green; *X*^2^ = 3.556, *df* = 1, *p* = 0.059).

There were no significant main effects, or interaction between taste and sex on the total number of pecks on red or green frogs (Table 2). Overall, chicks directed significantly more pecks at green than red frogs (green: 4.55 ± 0.6; red: 2.05 ± 0.4; *V* = 648, *p* < 0.002; Fig. 2D).

### Shape preference

#### Green sphere vs. green frog

**Fig. 3:**
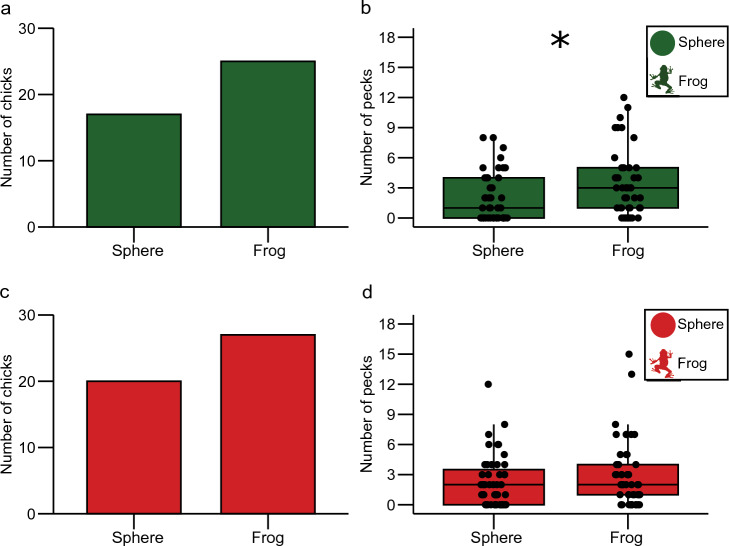
Shape preferences. **a** Number of chicks that directed their first choice to the green sphere and frog. **b** Total number of pecks on green sphere and frog. **c** Number of chicks that directed their first choice to the red sphere and frog. **d** Total number of pecks on the red sphere and frog. *Statistically significant differences

There were no significant main effects or interactions between taste and sex on the chicks’ first peck (Table 1). A similar number of chicks pecked first the green sphere or the frog (17 out of 42 chose spheres; *X*^2^ = 1.52, *df* = 1, *p* = 0.217; Fig. 3a).

Chicks pecked significantly more at frogs than spheres (frog: 3.5 ± 0.5; sphere: 2.05 ± 0.4; *t* = − 2.16, *df* = 41, *p* = 0.037; Fig. 3b). Chicks also showed a non-significant trend (*p* = 0.077; Table 2) to peck at both stimuli more after receiving water (2.9 ± 0.5) than quinine (2.61 ± 0.4).

#### Red sphere vs. red frog

There were no significant main effects or interaction between taste and sex on the first peck (Table 1). A similar number of chicks pecked first at red sphere or frog (20 out of 47 chose the sphere; *X*^2^ = 1.04, *df* = 1, *p* = 0.307; Fig. 3c). Likewise, the total number of pecks was similar between red spheres and red frogs (sphere: 3.08 ± 0.6; frog: 2.31 ± 0.3; *t* = − 1.13, *df* = 46, *p* = 0.261; Fig. 3d).

## Discussion

Here, we used a paired preference test for stimuli calibrated to the avian visual receptor responses to investigate the innate responses of domestic chicks to colour and shape. We show that dark-hatched, unfed, and visually naive domestic chicks avoid red stimuli in a preference test with green stimuli. Our experiment also revealed a preference for the shape of frogs over spheres, but only when the stimuli were green. When chicks experienced the bitter taste of quinine immediately before the preference test, the spontaneous preference for green stimuli increased in male chicks tested with frog-shaped objects. Our study is one of the first to test completely visually naive predators (though see Ham and Osorio 2007). We demonstrate that predators innately avoid colours typically associated with aposematism. Our results also provide evidence that experiencing other sensory cues can alter innate predispositions and that these affect predator responses to new food/prey during initial encounters. This is relevant to understanding the mechanism and adaptive significance of colour and shape preferences (e.g. identification of important resources and resource quality in inexperienced solitary animals), with added implications for the initial evolution of aposematism.

The preference that we report for green over red items (regardless of shape) partially aligns with previous evidence that young chicks prefer green over red insects (Gamberale-Stille and Tullberg 2001). However, in previous studies, chicks also showed no preference between red and green fruits (Gamberale-Stille and Tullberg 2001). In contrast, our study found that naive chicks prefer green over red spheres (resembling a fruit's typical shape). Our results suggest that newly hatched predators have innate predispositions that promote initial avoidance of any object with colours typically associated with aposematism, regardless of the stimulus shape, which could be later updated by experience.

In support of this idea, we found no initial preference for frogs over spheres when both stimuli were red, but older chicks in other studies preferred shapes of fruit over insects when the stimuli were both red (Zachar et al. 2008). Likewise, 9-day-old chicks selected red stimuli more than green stimuli, compared to visually naive day-old chicks that showed no preference for red over green (Ham and Osorio 2007). In addition, great tinamous in a field experiment preferred red spherical stimuli over red frogs. Since tinamous are sympatric with poison frogs, these preferences likely represent the behaviour of predators that had learned to avoid red frogs, or to be wary of them (Paluh et al. 2015). The responses of wild birds to red can also vary across seasons and years, and be influenced by the abundance of red food types in the environment, indicating a role of experience (Hartley 1953; Betts 1995; Teichmann et al. 2020).

An alternative explanation is that visual, textural, and olfactory differences between the stimulus materials (i.e. pastry spheres vs real insects in Gamberale-Stille and Tullberg (2001) and Zachar et al. (2008), and differences in the size of the paired stimuli, account for the differences to what we have found. Size differences in stimuli affect whether chicks make decisions based on chromaticity or luminance (Osorio et al. 1999). In our experiment, we controlled the visual conspicuousness, volume, olfactory cues, and textural cues of the stimuli. Another possibility is that early experience with social partners in other experiments explains the differences between our and other studies, as colour preferences of older birds are influenced by general experience (Miklósi et al. 2002).

A further possibility with this kind of paired preference tests that allow simple binomial statistics is an increased risk of type I and II errors. The forced choice nature of the test does not allow participants to express ‘no preference’. Experimental designs that feature replicate stimuli, and that require animals to investigate a set number of items (i.e. Rowe and Skelhorn 2005; Skelhorn and Rowe 2005), are better able to detect preference or avoidance behaviour. We encourage researchers to take this into account in designing experiments, as well as using controlled stimuli to investigate colour and shape preferences, and consider the animal’s age, experience levels and species/breed/strain. Standardising methodologies would, in the future, allow hypothesis-driven meta-analyses on the innate colour responses.

If our results represent an initial avoidance of red that is later updated by experience, this could suggest that birds are able to make adaptive food choices. In further support for the idea that innate colour and shape preferences are updated by experience, we found that male chicks that received the bitter taste of quinine before encountering the stimuli showed a stronger preference for green stimuli when both were frog-shaped. This aligns with research showing that chicks bias their attacks from red to green after experiencing quinine (Rowe and Skelhorn 2005; Skelhorn and Rowe 2005; Skelhorn et al. 2008). In contrast to males, the first choices of females that had received quinine did not differ between green or red frogs. Sexually dimorphic behavioural differences have previously been reported in chicks in various tasks (Vallortigara 1992; Miura and Matsushima 2012; Santolin et al. 2020; Rosa-Salva et al. 2023). Here, we provide the first tentative evidence of sex differences in responses to taste cues, but further replication would be beneficial.

We also found that naive birds preferred prey-shaped targets over round (fruit-shaped) ones, but only when they were green. This suggests that innate mechanisms can integrate colour and shape cues. We can hypothesise that chicks initially group our red stimuli into a category of general biological significance, but distinguish stimuli with colours less often associated with risk based on their shape. While our results show that naive chicks respond to differences in the stimuli’s shape, discriminating e.g. rounded vs legged objects, we expect finer discriminations to be experience dependent. We expect that, for naive chicks, both frogs and insects may belong to the general category of legged prey. Indeed, avoidance of red prey has been found using both insect-like (Gamberale-Stille and Tullberg 2001; Zachar et al. 2008) and frog-like prey stimuli (Paluh et al. 2015; as in the current study). This supports the idea that chicks’ innate avoidance responses are not restricted to a specific type of prey but rather encompass more general categories that have biological significance. This would align with what is found in studies of innate social behaviours. Innate preferences have been reported for elementary visual features typically associated with the presence of living creatures, such as self-propelled biological motion or the presence of face-like configurations, rather than for species-specific or even individual features of social companions (Rosa Salva et al. 2015; Di Giorgio et al. 2017; Rosa-Salva et al. 2018, 2019, 2023; Lemaire et al. 2022). This has been interpreted as evidence that the innate predispositions of naive domestic chicks are typically based on “coarse” representations of various objects categories (e.g. a self-propelling object of about a chick´s size will be approached as a social companion; see Rosa-Salva et al. 2021 for reviews). In agreement with that, classical neuroethological models (e.g. Ewert 1987) predict that animals will innately treat objects as potential food if they are small enough to be manipulated for ingestion, while objects of about the same size as the animal will be treated as potential social companions and even bigger objects as potential predators.

In conclusion, we show that chicks innately prefer green over red in their first encounters with potential food sources, regardless of their shape. These innate biases against colours typically associated with aposematism could increase the survival of conspicuous prey in the presence of naive predators that have yet to learn about the link between unprofitability and conspicuousness (Mappes et al. 2005). Our results also suggest that predators’ early life experiences and social systems can play a significant role in the evolution of antipredator defences in prey (see also Hämäläinen et al. 2021).

### Supplementary Information

Below is the link to the electronic supplementary material.

Supplementary file1 (DOC 75 KB)

## Data Availability

The datasets generated during and/or analysed during the current study are available in Edmond—the Open Research Data Repository of the Max Planck Society: https://doi.org/10.17617/3.ZHSGVS.
