# Supplementary material for: In paired preference tests, domestic chicks innately choose the colour green over red, and the shape of a frog over a sphere when both stimuli are green
Source: Anim Cogn. 2023 Aug 23;26(6):1973–83. doi: 10.1007/s10071-023-01821-x (PMC10769926; doi:10.1007/s10071-023-01821-x)
Supplement: Supplementary file 1 — Supplementary file1 (DOC 75 KB) [file 10071_2023_1821_MOESM1_ESM.doc]

**Supplementary Information**

**In paired preference tests, domestic chicks innately choose the colour green over red, and the shape of a frog over a sphere when both stimuli are green**

Francesca Protti-Sánchez^1^, Uwe Mayer^2^, Hannah M. Rowland^1^

^1^Max Planck Research Group Predators and Toxic Prey, Max Planck Institute for Chemical Ecology, Hans Knöll Straße 8, Jena, 07745, Germany

^2^Center for Mind/Brain Sciences (CIMeC), University of Trento, Piazza Manifattura 1, 38068 Rovereto, TN, Italy

**Resumen (Spanish)**

**En pruebas de preferencia pareadas, pollitos domésticos eligen de manera innata el color verde sobre el rojo y la forma de una rana sobre una esfera cuando ambos estímulos son verdes.**

Muchos animales expresan preferencias de color no aprendidas que dependen del contexto en el que las señales son encontradas. Estos sesgos de color pueden haber evolucionado en respuesta al sistema de señalamiento con el que se relacionan. Por ejemplo, muchos animales aposemáticos anuncian su falta de rentabilidad con señales de advertencia rojas. Los sesgos innatos de los depredadores contra estos colores de advertencia han sido sugeridos como una de las posibles explicaciones para la evolución inicial del aposematismo. Sin embargo, no está claro si las preferencias de color no aprendidas reportadas en varias especies son realmente un comportamiento innato o si se basan en experiencia previa. En este estudio examinamos las preferencias espontáneas de color y forma de pollitos domésticos (Gallus gallus) eclosionados en la oscuridad, sin alimentación previa y sin experiencia visual. En cuatro experimentos, les presentamos a los pollitos la posibilidad de elegir entre rojo (un color típicamente asociado con patrones de advertencia) o verde (un color asociado con presas crípticas palatables), esferas de volumen semejante (que representan una forma de fruta generalizada) o ranas (que representan una forma de animal aposemático). Los pollitos prefirieron de manera innata los estímulos verdes y evitaron el rojo. Los pollitos también prefirieron la forma de rana a la de esfera cuando ambos estímulos eran verdes. Sin embargo, no hubo preferencia por las ranas sobre las esferas cuando los estímulos eran rojos. Los pollitos machos que experimentaron un sabor amargo de quinina inmediatamente antes de la prueba de preferencia mostraron una mayor preferencia por los estímulos con forma de rana verde. Nuestros resultados sugieren que los pollitos recién eclosionados integran de manera innata señales de color y forma durante la toma de decisiones, y que esto se puede aumentar con otras experiencias sensoriales. El comportamiento innato y basado en la experiencia podría conferir una ventaja de adaptativa (i.e. fitness) a nuevas presas aposemáticas y favorecer la evolución inicial de una coloración llamativa


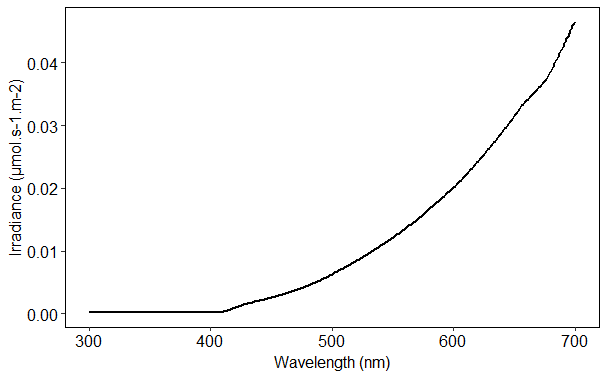


**Fig. S1.** Irradiance spectrum of the light used above the experimental cage.


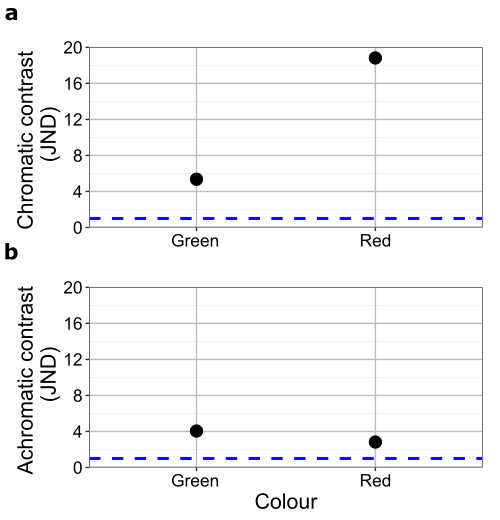


**Fig. S2.** Chromatic (a) and achromatic (b) contrast of stimuli colour (green and red) for the visual system of domestic chicks (see methods for further details about calculation). Dashed blue line represent the detection threshold (1 JND), meaning that values above this threshold are easily detectable for the observer.


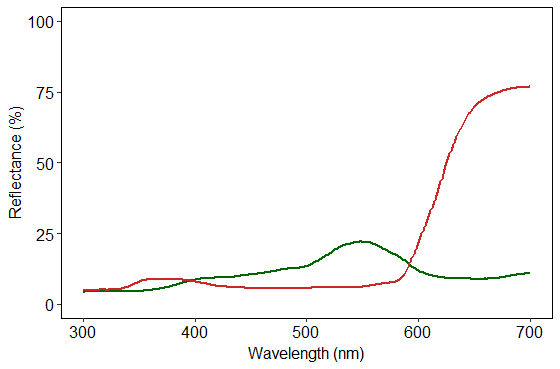


**Fig. S3.** Reflectance spectra of the paints used to colour the stimuli; red line=red paint, green line= green paint.

**Table S1.** Colourimetric variables from the paint used to colour the stimuli. Values obtained from the function summary() from the package pavo2 (Maia et al. 2018) used in R Studio v. 4.2.2 (R Core Team 2022).

| Colour measurement | Description | Green | Red |
| --- | --- | --- | --- |
| Hue | Wavelength of peak reflectance (H1) | 547 nm | 700 nm |
| Brightness | Average reflectance over all wavelengths (B2) | 10.97 | 20.62 |

**References**

Maia R, Gruson H, Endler JA, White TE (2018) pavo 2: new tools for the spectral and spatial analysis of colour in R

R Core Team (2022) R: A Language and Environment for Statistical Computing. R Found Stat Comput Vienna, Austria https://www.R-project.org
